# Synthesis of Antioxidant Nano Zero-Valent Iron Using FeCl_2_ and *Leucaena leucocephala* Leaves’ Aqueous Extract and the Nanomaterial’s Potential to Promote the Adsorption of Tartrazine and Nigrosine

**DOI:** 10.3390/ijms26125751

**Published:** 2025-06-16

**Authors:** Fernanda Maria Policarpo Tonelli, Christopher Santos Silva, Geicielly da Costa Pinto, Lucas Santos Azevedo, Jhenifer Cristina Carvalho Santos, Danilo Roberto Carvalho Ferreira, Pamela da Rocha Patricio, Giullya Amaral Cordeiro Lembrança, Luciana Alves Rodrigues dos Santos Lima, Clascídia Aparecida Furtado, Flávia Cristina Policarpo Tonelli, Adriano Guimarães Parreira

**Affiliations:** 1 Biotechnological Processes Laboratory, Federal University of São João del-Rei, Centro-Oeste Campus, Divinópolis 35501-296, MG, Brazil; christopher.silva.s@outlook.com (C.S.S.); geicicosta1212@gmail.com (G.d.C.P.); diuamaral@outlook.com (G.A.C.L.); flacristinaptonelli@gmail.com (F.C.P.T.); 2 Phytochemistry Laboratory, Federal University of São João del-Rei, Centro-Oeste Campus, Divinópolis 35501-296, MG, Brazil; azevedolucas07@gmail.com (L.S.A.); luarsantos@ufsj.edu.br (L.A.R.d.S.L.); 3 Department of Food Science, Federal University of Lavras (UFLA), Lavras 37200-900, MG, Brazil; jhenifercristinacs@gmail.com; 4 Nuclear Technology Development Center (CDTN), Belo Horizonte 31270-901, MG, Brazil; danilorcferreira@gmail.com (D.R.C.F.); clascidia@gmail.com (C.A.F.); 5 Chemistry Department, Minas Gerais State University (UEMG), Divinópolis 35501-170, MG, Brazil; pamela.patricio@uemg.br; 6 Protein Chemistry Laboratory, Federal University of São João del-Rei, Centro-Oeste Campus, Divinópolis 35501-296, MG, Brazil; aguiparreira@ufsj.edu.br

**Keywords:** green nano zero-valent iron, *Leucaena leucocephala*, dyes removal, antioxidant, green synthesis, nanotechnology, nigrosine, tartrazine

## Abstract

Synthetic dyes are commonly present in industrial wastewater and when discharged in water bodies without receiving a treatment capable of removing or destroying them, they turn into concerning water pollutants. These organic contaminants threaten living beings due to their toxicity, and some of them can even damage DNA. Consequently, in order to achieve sustainable development, it is necessary to develop eco-friendly tools that can efficiently manage this kind of pollution. In the present study the aqueous extract from the leaves of *Leucaena leucocephala* (an invasive plant species native to Mexico) was used to produce green nano zero-valent iron (nZVI). The nanomaterial was characterized (TEM, UV–vis, FTIR, SEM, EDS, XRD) and assayed regarding its antioxidant potential (DPPH test) and capacity to remediate the pollution caused by two dyes. It proved to be able to adsorb nigrosine (288.30 mg/g of nanomaterial) and tartrazine (342.50 mg/g of nanomaterial), and also displayed antioxidant activity (effective concentration to discolor 50% of the DPPH solution = 286.02 μg/mL). Therefore, the biogenic antioxidant nanoparticle proved also to be a possible nanotool to be applied to remediate water contamination caused by these synthetic dyes.

## 1. Introduction

When discharged directly into water from rivers, lakes or into the sea, inadequately treated or untreated industrial effluents cause serious problems, affecting living beings in a negative manner. These effluents are rich in synthetic dyes, commonly resistant to methodologies traditionally applied for water treatment due to their stable and complex structures. To worsen the problem, these dyes commonly present aromatic systems -N=N-, which makes it possible to classify them as azo dyes (these account for around 50% of dyes produced worldwide). These types of dyes exhibit poor fixation and are, consequently, present in large amounts in residual industrial water [1].

Tartrazine, an anionic dye commonly used in industries that produce cosmetics, pharmaceuticals and food, is an example of a dangerous azo dye. It can cause not only allergies, but also alterations in the central nervous system and DNA damage [2]. However, not only azo dyes can cause problems for humans. Nigrosine is also an anionic dye; however, it is not an azo one. This dye can be produced in an iron-like or copper atmosphere through an aniline hydrochloride, aniline and nitrobenzene mixture under warming [3]. Nigrosine is useful in the histology field and also in industries such as the textile one. Although it is not a mutagenic substance [4] it can still cause respiratory discomfort and induce allergic responses [5].

As a consequence of water pollution, these dyes can reach humans directly through drinking water, or indirectly through dietary habits of consuming, for example, aquatic species contaminated by these pollutants. In fact, these contaminants can bioaccumulate along the food chain, resulting in human exposure to higher doses [6]. In the presence of these pollutants, chemical and biochemical oxygen demands are increased, photosynthetic activity is impaired, and extensive mortality of aquatic organisms as a result of water eutrophication can occur [7].

Water is a vital resource, and of the available amount in the world, only less than 0.01% is drinkable [8]. So, it is urgent to remediate its pollution and focus on sustainability principles to prevent further contamination.

Nanoparticles (NPs) are materials with small dimensions (commonly in the range of 1 to 100 nm) that can be elegant solutions in the management of environmental contaminants, especially the green or biogenic versions, that are eco-friendly [9]. Not only sustainable but also cost-effective protocols can be developed to obtain nanotools, for example, from waste material, capable of removing pollutants (for example by efficiently adsorbing them) [9,10].

Iron NPs (FeNPs) are less toxic than NPs containing other metals such as Ti, Zn and Ag, and more economical when it comes to the synthesis protocols. They can be classified in groups depending on their chemical composition: Fe_3_O_4_ and Fe_2_O_3_ belong to the group of iron oxide nanoparticles; FeOOH is an iron oxide hydroxide; and the nano zero-valent iron (nZVI) is a nanoparticle that presents a core of Fe^0^ and a shell of iron hydroxides and oxides [11].

The nZVI presents advantageous features regarding adsorption of pollutants and hydrodynamic properties that favor its use in promoting the removal of contaminants from water. This nanomaterial can be produced through green protocols (avoiding the use of toxic reagents and the synthesis of harmful coproducts) to exhibit useful activities to act not only in the mentioned type of removal; it can also be a useful material in the medical field due to anticancer, antioxidant and antimicrobial activities, for example [12].

Plant extracts can be added as raw material to a source of iron atoms (like a solution of a salt containing the metal, such as FeCl_2_) to the production of green nZVI. The phenolics produced by vegetal species are important not only in redox reactions (acting as reductants) but can also act as capping agents to functionalize the nanomaterial produced, stabilizing its structure [13,14]. An interesting source of the plant material to be used in the synthesis of green nanomaterials are invasive species, as they are problematic species that threaten local biodiversity and impair crop yield in the agricultural sector [15]. In fact, the second place on the list of factors responsible for plant loss biodiversity worldwide is invasive species [16].

**Figure 1:**
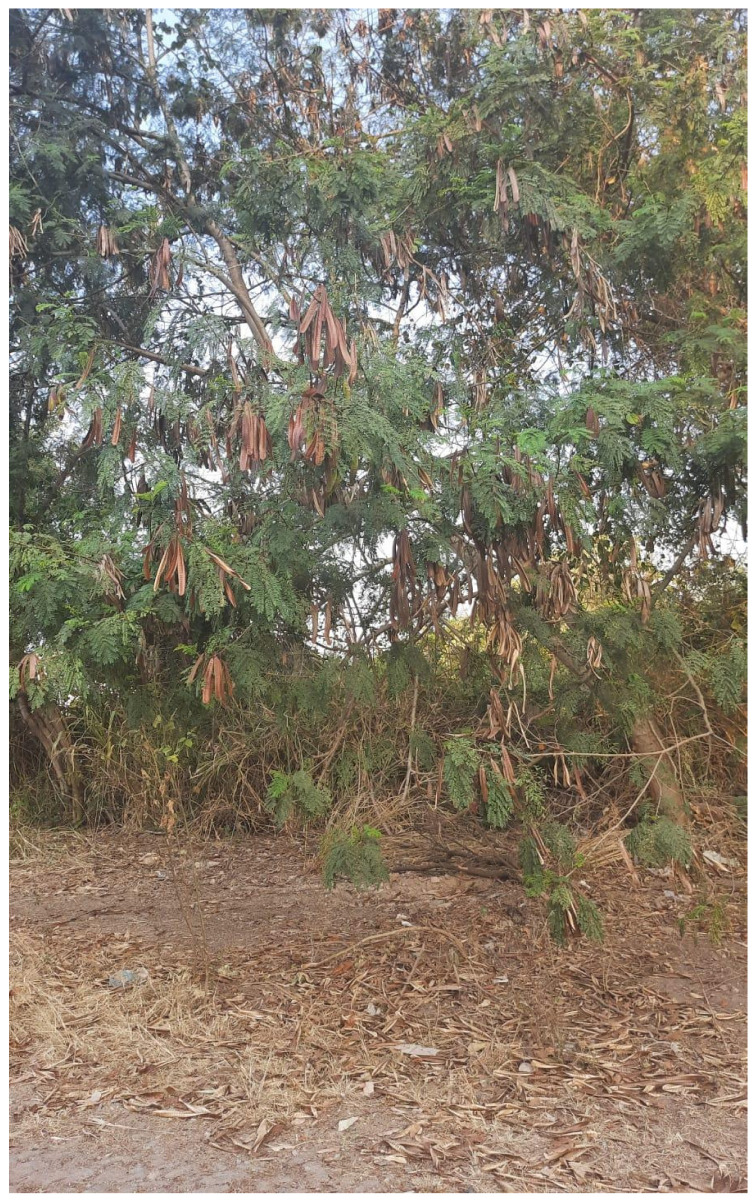
Leucena photo at the spot in which plant material was collected to perform this study.

The species popularly known as Leucena (*Leucaena leucocephala* (Lam.) de Wit) (Figure 1) is native to Mexico. In Brazil, for example, this leguminous N fixing species was introduced to be used to feed livestock and to help to recover the soil from deforested areas [17]. The seeds from this species can be used in the human diet due to the large content of proteins they exhibit [18]. However, this invasive species is problematic to agriculture and the problems caused by Leucena are not limited to Brazil. More than 125 countries in Asia, Africa, Central and South America, Europe and the Middle East, for example, are currently suffering the negative impacts of this weed species [16,18,19].

The extract of Leucena seeds could already be used to produce FeNPs; however, these nanoparticles differ from those of the present study because they were made of iron oxide, different to the nZVI used in the experiments reported here. The iron oxide particles were able to remediate the pollution associated with Congo red dye [20].

The present work is dedicated to developing a protocol that allows the synthesis of nZVI using the aqueous extract from the leaves of Leucena (containing an optimized amount of phenolics). The nanomaterial produced was investigated regarding its capacity to remove the pollutants nigrosine and tartrazine (without requiring any specific reduction agent or direct exposure to sunlight) from aqueous samples, and was also screened to assess its antioxidant potential.

## 2. Results

### 2.1. Influence of the Temperature in the Extraction of Phenolics from the Leaves of Leucena

The crude extract of the leaves from *L. leucocephala* suffered influence from the temperature when it comes to their content of phenolics, as presented in Table 1. The temperature that caused the extract to be richer in these substances was the 80 °C one: 329.94 ± 0.05 mg of gallic acid equivalent (GAE)/g of extract.

### 2.2. Influence of Time in the Extraction of Phenolics from the Leaves of Leucena

The extraction time exerted influence over the extraction of phenolics from powder material from the leaves of Leucena (Table 2). At the optimized extraction temperature (80 °C), maintaining the extraction for 20 min revealed to be a scenario that could favor the extraction of these substances, allowing the obtainment of a crude aqueous extract presenting 386.42 ± 0.32 mg GAE/g of extract.

### 2.3. Influence of the pH and the Proportion of FeCl_2_:Extract in the Potential of nZVI to Promote Removal of Dyes

The pH and the proportion of FeCl_2_:Extract influenced the potential of nZVI to act to mitigate the pollution caused by tartrazine and nigrosine in aqueous samples (Table 3, Table 4 and Table 5). The proportion 1:3 at pH 6.0 revealed to be the best scenario when it comes to the management of the pollution caused by both dyes, offering: 97.54% removal efficiency to tartrazine and 96.14% removal efficiency to nigrosine.

### 2.4. Characterization of nZVI

**Figure 2:**
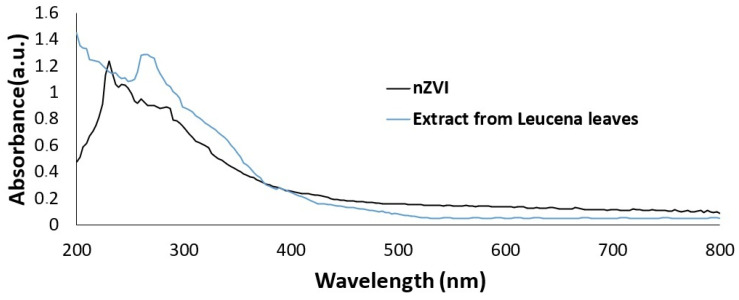
UV–vis spectroscopy results from Leucena leaves-based nZVIs and from the extract obtained from the leaves of Leucena and used to synthesize the nanomaterial.

The UV–vis spectroscopy was used to analyze the produced nZVI in the wavelength range of 200 to 800 nm. The maximum absorbance could be detected at 230 nm (Figure 2). This picture also presents the UV–vis spectrum from the extract obtained from the leaves of Leucena in the concentration used to produce the analyzed nanomaterial.

**Figure 3:**
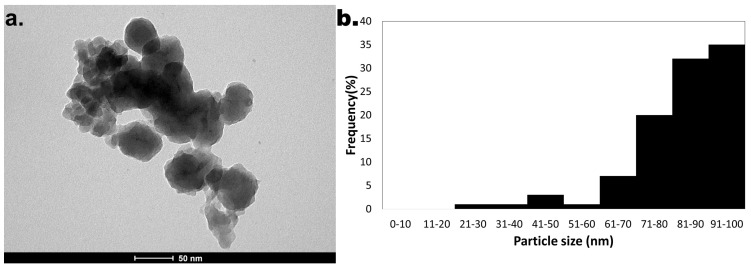
TEM analysis of nZVI: (**a**) Image of nZVI; (**b**) The profile of size distribution associated with the nanomaterial.

The average diameter of 83.0 ± 11.3 nm was exhibited by nZVIs and through transmission electron microscopy (TEM) analysis, it was possible to observe not only the size, but also the shape of the nanomaterial: a quasi-spherical one (Figure 3).

**Figure 4:**
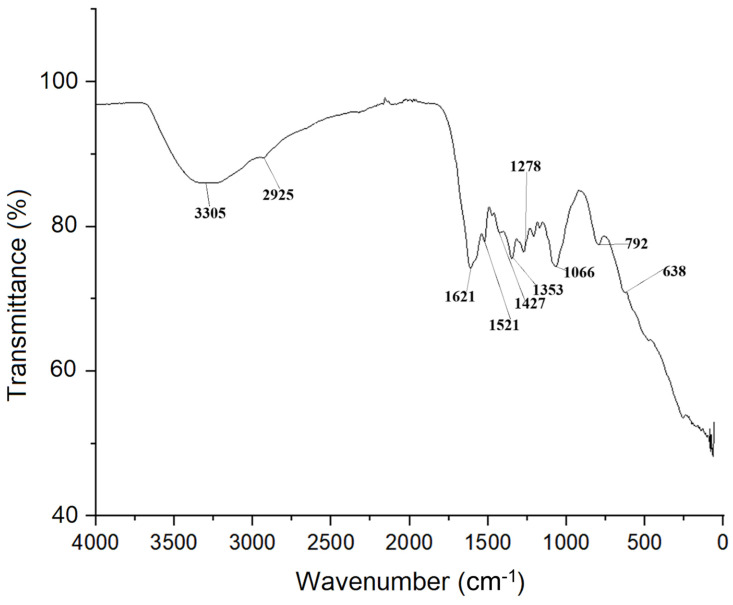
FTIR analysis of the synthesized nZVI.

The presence of functional groups in the nanomaterial’s surface was analyzed through Fourier Transform Infrared Spectroscopy (FTIR) (Figure 4). Among the absorption bands observed, it is possible to highlight the ones with a maximum at approximate wavenumbers of 3306 cm^−1^ (related to stretching vibrations from O–H of polyphenols); 2926 cm^−1^ (associated with aliphatic hydrocarbons’ C–H and CH_2_ vibrations); 1622 cm⁻^1^ (related to the C=O stretching vibration from carbonyl groups); 1522 cm^−1^ (associated with phenolics aromatic ring stretching vibration); 1427 cm^−1^ (related to bending vibrations from phenolic O–H); 1354 cm^−1^ (associated with ester C–O stretching); 1279 cm^−1^ (related to cyclic phenolics C–O asymmetric stretching); 1066 cm^−1^ (related to O–H deformation from alcohol or ethers); 793 cm^−1^ (associated with bending vibration from Fe–O–H); and 638 cm^−1^ (related to stretching vibration from Fe–O) [21,22,23,24,25].

**Figure 5:**
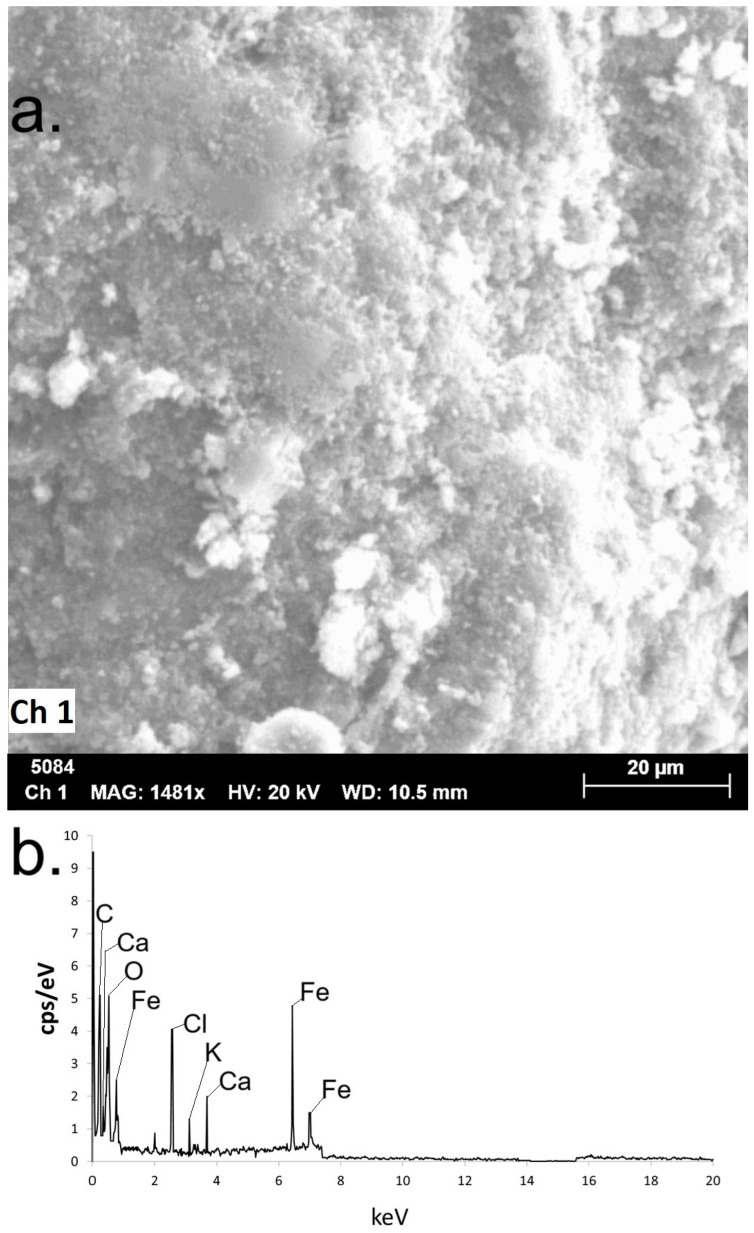
(**a**) SEM image of the synthesized nZVI. (**b**) EDS analysis of the produced green nZVI.

A scanning electron microscope (SEM) offered an image of the nZVI sample surface, confirming the quasi-spherical shape of the nanomaterial (Figure 5a). The electron scattering spectroscopy (EDS) revealed the presence of iron atoms, and signals referring to Cl, C and O could also be identified (Figure 5b). The Cl is present in the salt used to perform synthesis, and can also be a contribution of the plant material applied in the green protocol. The signals associated with O and C are a contribution of the polyphenol groups (also noticed in FTIR analysis), a consequence of capping the plant extract metabolites promoted on the surface of the nZVI [20,21,22,26]. We also identified signals associated with Ca and K (contribution of extracts from the leaves of Leucena) [27,28,29,30].

The hydrodynamic diameter of nZVI could be measured through scattering dynamic light (SDL) analysis: 610.40 ± 5.50 nm. Regarding size distribution, the polydispersity index was 0.249. When it comes to the average value of zeta potential, it was +22.30 ± 2.57 mV.

**Figure 6:**
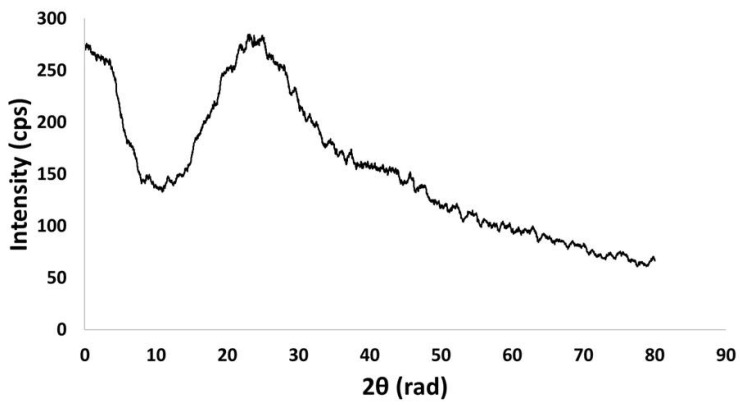
Diffractogram from the XRD analysis of the produced nZVI.

The X-ray diffraction (XRD) analysis of nZVI (Figure 6) does not suggest a crystalline material. The broad hump at approximately 2θ = 25° is due to organic structures from the capping process. The 2θ = 45° due to crystalline zero-valent iron (110) plane [22,24,31,32,33,34,35] is not possible to be detected in the diffractogram.

### 2.5. nZVI Removing Synthetic Organic Dyes from Aqueous Samples

**Figure 7:**
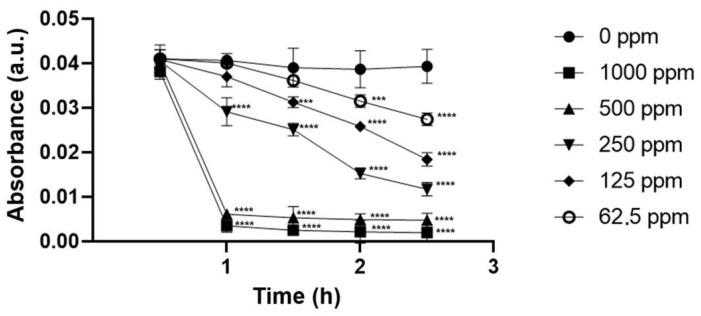
Tartrazine’s removal (pH = 6.0) performed by green nZVI (produced using the 1:3 proportion iron salt/extract) at five different concentrations. Statistically significant results compared to the control group are highlighted using asterisk (*** *p* < 0.001; **** *p* < 0.0005).

The eco-friendly nZVI produced at pH 6.0 could act in the removal of the pollution caused by tartrazine in water samples at the same pH. However, the efficiency of this process varied with the applied concentration of the nanomaterial (Figure 7).

**Figure 8:**
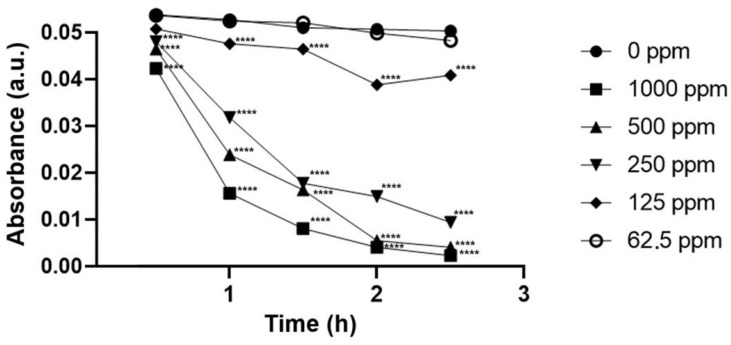
Nigrosine’s removal (pH = 6.0) performed by green nZVI (produced using the 1:3 proportion iron salt/extract) at five different concentrations. Statistically significant results compared to the control group are highlighted using asterisks (**** *p* < 0.0005).

When it comes to nigrosine, the pollution associated with this dye could also be remediated by the nanotool in a concentration-dependent manner at pH 6.0 (Figure 8).

At the maximum concentration tested, nZVI proved to be able to remove, after 2.5 h, 97.54% and 96.14% of the pollution caused by tartrazine and nigrosine, respectively.

### 2.6. Adsorption Kinetics

**Figure 9:**
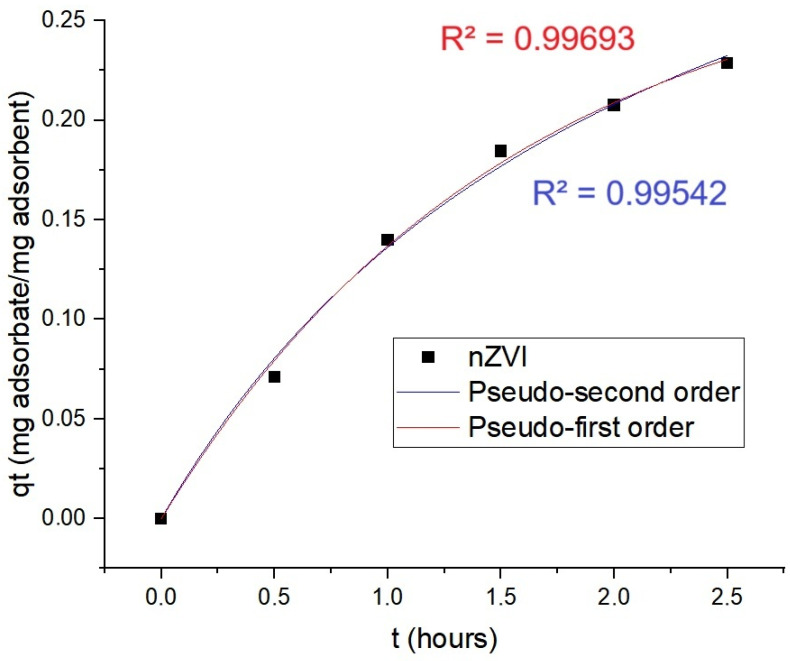
The nZVI absorption kinetics of nigrosine dye.

Figure 9 exhibits the results from adsorption kinetics involving the dye nigrosine. Adjustments involving pseudo-second order offered R^2^ = 0.99542 and the ones related to pseudo-first order offered R^2^ = 0.99693. The q_e_ for the best adjustment (pseudo-first order) was 288.30 mg/g of nanomaterial.

**Figure 10:**
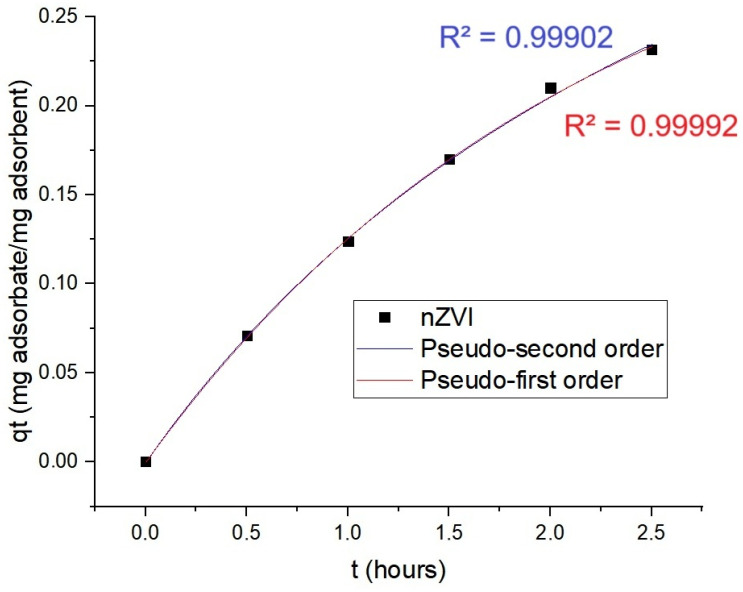
The nZVI absorption kinetics of tartrazine dye.

Figure 10 exhibits the results from adsorption kinetics involving the dye tartrazine. Adjustments involving pseudo-second order offered R^2^ = 0.99902 and the ones related to pseudo-first order offered R^2^ = 0.99992. The q_e_ for the best adjustment (also pseudo-first order) was 342.50 mg/g of nanomaterial.

### 2.7. Antioxidant Activity Assay

Table 6 exhibits the results of the antioxidant assay performed through the methodology involving 1,1-Diphenyl-2-picrylhydrazyl (DPPH). The nanomaterial presented antioxidant activity; however, it was not capable of surpassing the antioxidant activity of butylated hydroxytoluene (BHT). The effective concentration to discolor 50% of the DPPH solution for the nanomaterial was 286.02 μg/mL, while the one for BHT was 166.66 μg/mL.

## 3. Discussion

Benjakul and coworkers studied the influence of the solvent in the extraction of phenolics from *L. leucocephala* seeds and observed that water at 100 °C offered 545.00  ±  46.80 mg GAE/g of extract; in 80% ethanol and 100% ethanol, phenolics dosage was approximately 26.4% and 44.5% higher than this value, respectively [36]. So, in diminishing ethanol’s presence in the solvent, the amount of phenolics also diminishes. It occurs until it reaches a scenario of ethanol’s absence, which means that the solvent used in extraction is only water. Consequently, the amount of phenolics extracted using water is commonly inferior to the one obtained when using ethanol. Regarding the leaves of Leucena, the 90% ethanolic extract obtained by Singsai and coworkers contained 642.94 ± 183.4 mg GAE/g of extract: an amount that surpassed the one from fruits and seeds [37]. In the present study, the extract obtained from the leaves of Leucena using water at 80 °C presented a phenolics dosage of 386.42 ± 0.32 mg GAE/g of extract: an amount approximately 39.9% smaller than the one reported previously for the 90% ethanolic extract. Consequently, it is in accordance with the literature data.

Regarding the proportion FeCl_2_:Extract, the one that offered nZVI with the best performance in dealing with the pollution caused by the dyes was 1:3. Increasing the amount of plant extract to obtain the proportion 1:4 did not result in a better performance in pollution management. In the green synthesis, secondary metabolites from plants can act as capping agents. However, an increase in the amount of those agents does not always result in a better performance by the functionalized nanomaterials. As observed by Campisi and coworkers, an adverse impact can be provoked by them depending on variables such as spatial arrangement and coverage degree, as they may impair the nanomaterial’s ability to offer the intended activities [38]. In adsorption, for example, excessive capping can impair interactions between the nZVI and the dyes.

In this study, the synthesis of nZVI caused a change in the reaction mixture’s color to a dark one. This change in color is due to the oscillation of conduction band electrons from iron that goes from cationic form to Fe^0^. It results in a surface plasmon resonance band that can be detected through UV–vis analysis, indicating a successful synthesis. In this study, besides observing the change in color, UV–vis analysis of the nanomaterial revealed the expected band containing the absorbance maximum. It could be noticed at 230 nm, which is in accordance with the literature. This position of the absorbance maximum is in the region where zero-valent iron nanoparticles are expected to exhibit it [22]. It is also important to highlight that the plant extract, in this work, presented a band containing the maximum absorbance at 269 nm, which is in accordance with the results obtained by Raju and coworkers [39]. In the present study, it is possible to notice that the spectrum from clean nZVI, after the three washing cycles, is absent in this plant material-associated band.

The size distribution and shape of the nanomaterial produced in this work could be observed through TEM, and SEM allowed the observation of the nanomaterial’s surface. TEM analysis allowed the measurement of particles’ diameter and the assessment of an average diameter of 83.0 ± 11.3 nm. A quasi-spherical shape was also observed, and these particles were revealed to be similar in shape and size, for example, to the nZVI produced by Guo and coworkers using extracts obtained from grape seeds [40].

The FTIR analysis performed in this study revealed not only the presence of elements bound to iron (bending vibration from Fe–O–H and stretching vibration from Fe–O) but also groups from plant secondary metabolites, specifically phenolic acids and their derivatives: stretching vibrations from O–H, aliphatic hydrocarbons’ C–H and CH_2_ vibrations, C=O stretching vibration, aromatic ring stretching vibration, bending vibrations from O–H, ester C–O stretching, cyclic phenolics C–O asymmetric stretching, and O–H deformation. It is common that those metabolites act as capping agents in green synthesis, as previously mentioned, functionalizing the nZVI’s surface [21,22,24,25].

The presence of iron atoms in the nanomaterial could also be confirmed by the EDS analysis performed in this work, which also confirmed the presence of C and O from plant metabolites [20,21,22,26]. The Cl present in the salt used in the synthesis was also detected, as well as Ca and K [27,28,29] from the plant extract. The dry matter of the leaves from Leucena is rich in these metals; it contains 2.8% calcium and 1.78% potassium [30].

The SDL analysis from this study revealed a hydrodynamic diameter of 610.40 ± 5.50 nm. This value, more than seven times larger than the diameter measured through TEM, is an expected result. The latter allows size determination of individual particles within aggregates, unlike SDL [41]. Regarding size distribution, the polydispersity index observed in the present work was 0.249. This value between 0.1 and 0.25 is indicative of a narrow size distribution, resulting in homogeneous particles [42,43]. When it comes to the average value of zeta potential, it was +22.30 ± 2.57 mV, a value that neither can be used to classify the nZVI as approximately neutral (between −10 and +10 mV) nor to classify the material as strongly cationic (more than +30 mV) [44]. The nanoparticles can be considered as non-concerning when it comes to toxicity due to uncontrolled agglomerate formation [45].

The X-ray diffraction (XRD) analysis (Figure 6) of nZVI revealed a pattern similar to the ones observed in green nZVI previously produced by other research teams using various bio-based raw materials. The broad hump at approximately 2θ = 25° is due to organic structures from the capping process that occurs during green synthesis. A crystalline nZVI needs to present a peak at approximately 2θ = 45°, associated with the zero-valent iron (Fe^0^) (110) plane [24]. However, in green nZVI, when a large amount of organic structures from the bio-based material is present, it is possible that the 2θ = 45° is not visible in the diffractogram. Eslami and coworkers [22] and Wang and coworkers [33] produced nZVI, presenting an XRD that does not present an evident 2θ = 45°, but a broad hump at approximately 2θ = 25°. It is a characteristic of an amorphous nZVI, as the one produced in this work.

When it comes to the assay of the dye removal, it is important to highlight that the efficiency offered by the nZVI in the present work through adsorption is independent from the addition of reducing agents (such as NaBH_4_), special lamps (such as xenon) or direct exposure to sunlight.

Among the conditions analyzed in this study, the best conditions to obtain an aqueous extract, rich in phenolics, from the leaves of *L. leucocephala* were as follows: 80 °C and 20 min. These conditions are in accordance with a previous study this research group performed involving the obtainment of aqueous extracts from the leaves of *L. leucocephala*, stem and fruits to produce active green silver nanoparticles. After new plant material acquisition, processing and extraction, the temperature of 80 °C and the extraction time of 20 min continued to be the ones that offered the higher amount of phenolics in the leaves’ aqueous extract [46]. However, in this previous study, the influence of the synthesis pH in the potential of the nanomaterial to promote dye removal was not investigated.

It is already known that pH influences, for example, nZVI’s mean aggregates size; the aggregates formed at pH = 7 are larger than the ones produced at pH = 5.0, for example [47]. However, the pH can also influence the material’s zeta potential. Acidic pHs favor a positive surface charge, contributing to the adsorption of anionic structures such as phosphate [48]. In the present study, at a pH = 6.0 (deionized water), the zeta potential of the nanomaterial was +22.30 ± 2.57 mV, which could have favored the electrostatic adsorption of the two anionic dyes assayed in this study (offering maximum percentages of removal: 97.54% (tartrazine) and 96.14% (nigrosine)). The pH = 6.0 also offered the maximum efficiency (96.8%) of methylene blue’s removal by nZVI produced using *Ricinus communis* aqueous seed extract [24]. In the present study it was possible to notice that an increase in pH really impaired this type of removal (electrostatic adsorption), which is in accordance with the studies of Almeelbi and coworkers and Lin and coworkers [47,48]. However, the immediate idea would be to imagine that a pH lower than 6.0, such as 5.0, would favor more the removal of anionic dyes. But observing the results presented in this study, this is not true. In accordance with the work of Altuntas and Debik, a pH lower than the one that offered the best removal performance, as well as a pH higher, impaired the process. These scientists studied the management of DDT’s by nZVI and concluded that at pH = 6.5, the qmax coefficient for Langmuir isotherm was 23.71; at pH = 4.0 it was 23.38, and at a pH of 10 it was 22.26 (both values smaller than the one obtained at pH = 6.5) [49]; that is, both a pH lower than or higher than the optimum pH negatively impacts the removal process. This can be due to an increase in the positive zeta potential induced by more acid pHs. Higher zeta potential favors a scenario in which the repulsive forces exceed the attractive ones, which can influence the establishment of interactions of substances with the nanomaterial’s surface [50,51]. However, when comparing maximum percentage of removal from the more acidic pH (pH = 5.0–87.47% (tartrazine) and 93.78% (nigrosine)) to the one from a more basic one (pH = 7.0–63.52% (tartrazine) and 63.17% (nigrosine)), the first scenario offered better results following the expected behavior regarding exhibiting a nZVI’s more positive surface charge [48].

Iron nanoparticles have already been produced using Leucena’s material. The seeds were the raw material for the synthesis of Fe_3_O_4_ nanoparticles. The plant extract was obtained using a 3 M NaCl solution to generate a material that could remove 90% of the pollution caused by the synthetic dye Congo red [20]. The nZVI could also be produced using the extract from the seeds to promote a Fenton-like catalysis to deal with the pollution caused by the dye methylene blue; 32% of the dye (10 ppm) could be degraded by the nanomaterial (40 mg/L) after 3 h [52].

When it comes to the kinetics of dyes’ adsorptive removal observed in the experiments performed in this study, it adjusted better to the pseudo-first order than to the pseudo-second one for both dyes (tartrazine and nigrosine). The q_e_ for these nanotools able to promote dyes’ removal surpassed the one observed by Kristianto and coworkers for the Leucena seeds-based iron nanoparticles (approximately 43 mg/g) that presented a pseudo-second order kinetics in Congo red’s removal [20]. The pseudo-first order model also adjusted to the green nZVI produced by Abdelfatah and coworkers, from *Ricinus communis* seeds extract to remediate methylene blue. However, the nZVI produced in the present study from Leucena surpassed the capacity of the one from that study to remediate the pollution caused by the dyes. A q_e_ of 288.30 mg/g was exhibited for nigrosine adsorptive removal and a q_e_ of 342.50 mg/g for tartrazine; for methylene blue, q_e_ was 8.78 mg/g [24].

Regarding the antioxidant activity, it became a desirable feature once oxidative stress was related to various diseases such as cancer and Parkinson’s [53]. Antioxidants can assist in the fight against free radicals, contributing to humans’ health and life quality. An interesting aspect in the medical field is that nanoparticles can present this potential combined with increased bioavailability when compared to regular antioxidants, offering also the opportunity of functionalization aiming at controlled release performing a targeted delivery.

The use of phenolics to generate the nZVI favors this activity once these plant secondary metabolites commonly present this property [54]. The lower the effective concentration of a substance necessary to discolor 50% of the DPPH solution, the higher the antioxidant property of the nanomaterial. The nZVI produced in this study presented antioxidant activity, with DPPH inhibition ranging from 0.66% to 58.66% at concentrations of 1 to 500 μg/mL, which is in accordance with the literature. The nZVI produced using *Myrtus communis* leaf extract exhibited antioxidant activity (effective concentration to discolor 50% of the DPPH solution = 11.42 ± 0.96 μg/mL) that surpassed the one from the plant extract (effective concentration to discolor 50% of the DPPH solution = 12.5 ± 0.65 μg/mL); the contribution of phenolics (that guided green synthesis and functionalized the nanomaterial) was highlighted [22]. *Musa coocinea* peel extract could already be used to produce nZVI that exhibited scavenging activity for DPPH radicals that varied from 32 to 47.86% as the concentration of the nanomaterial varied (200, 400, 600, 800 and 1000 μg/mL) [55]. Iron NPs synthesized using *Argemone mexicana* as raw material exhibited 97.52% antioxidant activity in DPPH tests at a concentration of 1 mg/mL [56]. The nZVI produced from *Madhuca indica* showed antioxidant potential at concentrations of 200 to 1000 μg/mL, with scavenge free radicals (DPPH) from 49.16% to 81.53% [57]. However, even nZVI produced through non-green protocols have already proved to present antioxidant activity. At low concentration (such as 250 mg/L) in *Leonurus cardiaca*, the antioxidant activity of nZVI (effective concentration to discolor 50% of the DPPH solution = 44.51–40.61 μg/mL) surpassed the one from Fe_3_O_4_ (effective concentration to discolor 50% of the DPPH solution = 58.01–52.16 μg/mL) [58].

As future perspectives of this study, the reuse of nZVI will receive attention as well as its toxicity. The latter aspect needs to be explored initially in vitro over seed germination and development, human cells, and also cells from species that live in water bodies (such as fishes). Later, in vivo studies involving, for example, zebrafish and mice can help to enhance comprehension regarding not only toxicity but also distribution and elimination of these particles by these organisms.

## 4. Materials and Methods

### 4.1. Collecting and Preparing the Synthesis Bio-Based Raw Material

The leaves of *L. leucocephala* were collected in Minas Gerais state, Brazil, in Divinópolis city, at a region presenting the geographic coordinates 20°8′7″ S and 44°53′49″ W. In that location the plant species had already been collected previously for the development of another study; therefore, the voucher specimen was already deposited (number 58919 at the Agricultural Research Company of Minas Gerais—EPAMIG: Belo Horizonte, Minas Gerais, Brazil—PAMG herbarium) [46]. After cleaning the plant material, it was dried at 40 °C (until mass stabilization) in an oven (Ethik Technology; Vargem Grande Paulista, Brazil). The pulverization of this dried raw material was performed using a knife mill (Tecnal; Piracicaba, Brazil) and the powder obtained was stored.

### 4.2. Extraction at Different Temperatures

The stored dry leaves (10 g) were submitted to extraction using deionized water (100 mL) as solvent. Initially the ultrasonic bath Soniclean 2PS (Sanders Brasil; Santa Rita do Sapucaí, Brazil) was programmed to offer a 30 min extraction; however, five different extracts were obtained varying the temperature, increasing in 20 degrees Celsius increments (°C) from 20 °C to 100 °C. The crude extracts obtained were filtered, dried in a rotary evaporator IKA-HB10 digital (IKA; Shanghai, China) and stored (−4 °C).

### 4.3. Dosage of Phenolics

An adapted version of the colorimetric Folin–Ciocalteu method [59] was performed to assess the dosage of phenolics in the aqueous extracts. An analytical curve was generated using gallic acid 0.2 mg/mL (Sigma Aldrich; Duque de Caxias, Brazil) as a standard (5, 10, 20, 30 and 40 μg/mL). In triplicate the samples were added to water (250 μL) and received 2250 μL of Folin–Ciocalteu reagent (99.5%) (Sigma Aldrich; São Paulo, Brazil). The absorbance (750 nm) was verified after 30 min using a Genesys 180 UV–vis spectrophotometer (Thermo Fischer; São Paulo, Brazil). The absorbance values were then converted into mg of GAE/g of extract. The statistical analysis of the results was performed through two-way ANOVA with Tukey’s post-test (GraphpadPrism 7.00 software).

### 4.4. Extraction at Different Time Intervals

The stored dry leaves (10 g) were submitted to extraction using deionized water (100 mL) as solvent. Initially the ultrasonic bath Soniclean 2PS (Sanders Brasil; Santa Rita do Sapucaí, Brazil) was programmed to offer a temperature of 80 °C (the temperature that offered the richest extract when it comes to phenolics); however, five different time intervals were assayed, increasing in 10 min increments from 10 min until it achieved 1 h. The crude extracts obtained were filtered, dried in a rotary evaporator (IKA-HB10 digital; Shanghai, China), stored (−4 °C) and later submitted to the dosage of phenolics as described in Section 4.3.

### 4.5. nZVI Synthesis at Different pHs

A solution of 0.1 M of Fe^2+^ was prepared using tetrahydrated FeCl_2_ (Exodo Científica; Sumaré, Brazil). The crude extract containing the highest amount of phenolics was added to this solution in order to achieve, *v*/*v*, the following proportions of FeCl_2_ solution/extract: 1:1, 2:1, 1:2, 3:1, 3:2, 1:3, 2:3 and 1:4. The mixtures were submitted to agitation using a magnetic stirrer IKA C-MAG HS 7 (IKA; Campinas, Brazil) and drops of NaOH 1 M were added until the pH was adjusted to 5.0, 6.0 or 7.0. After 45 min the products were washed 2 times using deionized water through centrifugation (NT800—Nova Técnica; Piracicaba, Brazil): cycles of 20 min at 14,000 rpm. A supernatant transparent to the naked eye was obtained; however, when subjected to UV–vis spectroscopy, it still presented a subtle band (between 260 and 280 nm) referring to that from the plant extract spectrum. Then, a new washing step was performed to offer a supernatant absent in plant extract material. At 70 °C the nZVI samples were then dried.

### 4.6. The Removal of Tartrazine and Nigrosine by nZVI

Solutions (16 mg/L) of each the synthetic organic dyes tartrazine (Exodo Científica; Sumaré, Brazil) and nigrosine (Exodo Científica; Sumaré, Brazil) were prepared to be used in the dyes’ removal assay in 96-well plates [46]. Each nZVI sample produced at a certain pH was assayed in 5 different concentrations, in triplicate. To achieve these concentrations the maximum concentration assayed was prepared and the pH was adjusted to the one of synthesis (5, 6 or 7) using drops of NaOH and a pHmeter. This solution was submitted to serial dilution (2 being the dilution factor) to obtain the other ones that also underwent pH adjustment to the one from nZVI synthesis (5, 6 or 7). The concentration of the dyes was not varied in the experiment; they were assayed at 10 mg/L [60].

In the final template of each ELISA plate, the following triplicates were present: (1) water; (2) dye (always with a final concentration of 10 mg/L); (3) NPs at 1000 ppm in water; (4) NPs at 1000 ppm and dye; (5) NPs at 500 ppm in water; (6) NPs at 500 ppm and dye; (7) NPs at 250 ppm in water; (8) NPs at 250 ppm and dye; (9) NPs at 125 ppm in water; (10) NPs at 125 ppm and dye; (11) NPs at 62.5 ppm in water; and (12) NPs at 62.5 ppm and dye.

After an initial adsorption time of 30 min, the test plate was read every 30 min for 2.5 h (0.5, 1.0, 1.5, 2.0 and 2.5 h) at 560 nm (for nigrosine) and 427 nm (for tartrazine) using a PowerWave XS2/US ELISA spectrophotometer (Biotek; Santa Clara, CA, USA). The statistical analysis of the results was performed through two-way ANOVA with Tukey’s post-test (GraphpadPrism 7.00 software) [46].

### 4.7. nZVIs’ Characterization

The nanomaterial produced at the optimized temperature, time interval, proportion FeCl_2_:extract and pH, and that consequently offered the best removal performance, was submitted to characterization. The characterization analysis involved EDS, SEM (STEM-FEG-model Clara—Tescan; Brno, Czech Republic, with Quantax EDS—Bruker; Billerica, MA, USA), FTIR (MB102—ABB Bomem Inc.; Québec, QC, Canada), SDL and Zeta potential (at pH 6.0—Litesizer DLS 500—Anton Paar; Graz, Austria), TEM (Tecnai^TM^ G^2^ Spirit Biotwin 120 kV-FEI Company; Hillsboro, OR, USA), UV–vis spectroscopy (200–800 nm) (Genesys 150-Thermo Scientific; São Paulo, Brazil) and XRD (Rigaku D\Max Ultima III—Rigaku; Tokyo, Japan).

### 4.8. Adsorption Kinetics Assay

An amount of 25 mg of nZVI that exhibited the best dye-removal performance was added to 3 mg of each dye and water was added to complete 2.5 mL. The initial equilibrium was achieved through agitation for 30 min (25 °C) and the content was divided into tubes containing 500 μL each. One tube to each dye was submitted to centrifugation to allow the nZVI to form a pellet and the supernatant was analyzed using a Genesys 180 UV–vis spectrophotometer (Thermo Scientific; Saõ Paulo, Brazil) at 560 nm (for nigrosine) and 427 nm (for tartrazine). The other tubes were kept under agitation and every 30 min one tube of each dye was submitted to the centrifugation/analysis step mentioned. The total time of agitation was 2.5 h. The rates at which the dyes adsorbed onto nZVI were analyzed by applying pseudo-first- and pseudo-second-order equations [61].

The kinetic analysis was performed using OriginPro 10.1.0.170. Equation (1) was used to pseudo-first order and Equation (2) to pseudo-second order.

q_t_ = q_e_ (1 − e^−kt^) (1)

q_t_ = (kq_e_^2^t)/(1 + kq_e_t) (2)

In these equations q_t_ refers to the adsorption capacities at time t, k is the rate constant, q_e_ is the adsorption capacity at equilibrium and t is the time [62].

### 4.9. Antioxidant Assay

The DPPH (free radical) method [63] was applied to assess the characterized nZVI sample’s antioxidant potential. An adapted version, to be performed in microplates, involved an ethanolic solution of DPPH (0.002% *w*/*v*) from which 150 μL were added to each well of the test plate. Samples of nZVI and of BHT (positive control) (75 μL) were prepared to be assayed in triplicate in each of the following concentrations: 1, 10, 100, 250, and 500 μg/mL. For the negative control, ethanol was used. After adding samples, the 96-well plate was covered and kept in dark at 25 °C for 30 min. Then, the absorbance was assessed (517 nm) using a PowerWave XS2/US ELISA spectrophotometer (Biotek; Santa Clara, CA, USA).

To obtain the percentage of DPPH inhibition, the following equation (Equation (3)) was used [64]:

% DPPH inhibition = [1 − (Aa/Ab)] × 100(3)

This equation involves the absorbance of the sample (Aa) from the DPPH solution (Ab). Probit analysis [65] allowed the calculation of the effective concentration to discolor 50% of the DPPH solution.

## 5. Conclusions

The aqueous extract from the leaves of Leucena is capable of promoting the synthesis of eco-friendly nZVI. In this study, at pH 6.0, the mixture involving the largest amount of richest-in-phenolics extract (in comparison to the amount of FeCl_2_ solution present in the reaction pot) offered the best performance in adsorbing the dyes nigrosine and tartrazine. Consequently, this plant (which is a problem to the agriculture of countries in which *L. leucocephala* is present as an invasive species) can be applied in a sustainable manner in the nanotechnology field to assist in water pollution management.

The monodispersed nZVI displayed an amorphous structure in XRD and appeared as quasi-spherical under TEM analysis. FTIR revealed the presence of some functional groups from plant extract biomolecules capping the nanostructure that presented positive surface charge (which favored the interaction with the anionic dyes assayed).

Besides the potential to act as nanoremediators, promoting anionic dyes adsorption, the nZVI presented antioxidant activity against DPPH (free radicals).

As future perspectives, the possibility to reuse nZVI and the toxicity of the nanomaterial will be investigated, as well as the increase in the scale of its synthesis.

## 6. Patents

The work reported in this manuscript resulted in a patent deposit in Brazil. The INPI deposit number is BR 10 2025 008313 2.

## Figures and Tables

**Table 1:** Influence of the extraction temperature in crude Leucena leaves extract’s phenolics dosage.

Temperatures (°C)	Phenolics (mg GAE/g of Extract)
20	232.95 ± 0.02
40	243.52 ± 0.05
60	253.07 ± 0.03
80	329.94 ± 0.05 ***
100	262.25 ± 0.02

*** Indicates a statistically significant result associated with a *p* value inferior to 0.001 (*p* < 0.001).

**Table 2:** Influence of the extraction time in crude Leucena leaves extract’s phenolics dosage.

Time (Min)	Phenolics (mg GAE/g of Extract)
10	278.59 ± 0.05
20	386.42 ± 0.32 **
30	375.22 ± 0.09
40	380.34 ± 0.25
50	382.94 ± 0.05
60	381.19 ± 0.26

** Indicates a statistically significant result associated with a *p* value inferior to 0.05 (*p* < 0.05).

**Table 3:** nZVI acting in the removal of dyes at pH 5.0.

Proportion FeCl_2_: Extract	Removal of Tartrazine (%)	Removal of Nigrosine (%)
1:1	37.21	43.27
2:1	20.63	28.12
1:2	61.25	67.37
3:1	19.35	13.15
3:2	42.27	56.35
1:3	87.47	93.78
2:3	52.35	62.15
1:4	82.42	90.12

**Table 4:** nZVI acting in the removal of dyes at pH 6.0.

Proportion FeCl_2_: Extract	Removal of Tartrazine (%)	Removal of Nigrosine (%)
1:1	40.23	42.89
2:1	27.32	20.38
1:2	65.12	68.27
3:1	17.21	19.15
3:2	49.23	49.28
1:3	97.54	96.14
2:3	65.17	65.64
1:4	94.59	92.14

**Table 5:** nZVI acting in the removal of dyes at pH 7.0.

Proportion FeCl_2_: Extract	Removal of Tartrazine (%)	Removal of Nigrosine (%)
1:1	33.15	32.45
2:1	18.32	15.36
1:2	55.26	49.84
3:1	14.21	13.51
3:2	42.02	45.35
1:3	63.52	63.17
2:3	58.25	53.46
1:4	61.58	61.35

**Table 6:** Antioxidant potential of nZVI.

nZVI Concentration (μg/mL)	nZVI % of Inhibition	BHT % of Inhibition
1	0.66 ± 0.05	5.06 ± 0.11
10	6.39 ± 0.12	6.06 ± 0.24
100	21.81 ± 0.15	40.19 ± 0.17
250	46.18 ± 0.24	71.04 ± 0.05
500	58.66 ± 0.48	91.30 ± 0.08

## Data Availability

Data is contained within the article.
